# Psychotherapists’ Challenges With Online Therapy During COVID-19: Concerns About Connectedness Predict Therapists’ Negative View of Online Therapy and Its Perceived Efficacy Over Time

**DOI:** 10.3389/fpsyg.2021.705699

**Published:** 2021-07-22

**Authors:** Vera Békés, Katie Aafjes-van Doorn, Xiaochen Luo, Tracy A. Prout, Leon Hoffman

**Affiliations:** ^1^Ferkauf Graduate School of Psychology, Yeshiva University, Bronx, NY, United States; ^2^Department of Counseling Psychology, Santa Clara University, Santa Clara, CA, United States; ^3^New York Psychoanalytic Society & Institute, New York, NY, United States

**Keywords:** COVID-19, challenges, therapists, online therapy, therapeutic relationship

## Abstract

Therapists’ forced transition to provide psychotherapy remotely during the COVID-19 pandemic offers a unique opportunity to examine therapists’ views and challenges with online therapy. This study aimed to investigate the main challenges experienced by therapists during the transition from in-person to online therapy at the beginning of the pandemic and 3 months later, and the association between these challenges and therapists’ perception of the quality of the relationship with their online patients, and therapists’ attitudes and views about online therapy and its efficacy at these two timepoints. As part of a large-scale international longitudinal survey, we collected data from 1,257 therapists at two timepoints: at the start of COVID-19, when many therapists switched from providing in-person therapy to online therapy, as well as 3 months later, when they had had the opportunity to adjust to the online therapy format. At both timepoints, therapists reported on perceived challenges, quality of working alliance and real relationship, attitudes toward online therapy, and their views on online therapy’s efficacy compared to in-person therapy. Factor analysis of individual survey items at both timepoints identified four different types of challenges among this therapist sample: Emotional connection (feeling connected with patients, reading emotions, express or feel empathy), Distraction during sessions (therapist or patient), Patients’ privacy (private space, confidentiality), and Therapists’ boundaries (professional space, boundary setting). Older and more experienced therapists perceived fewer challenges in their online sessions. At baseline, all four types of challenges were associated with lower perceived quality of the therapeutic relationship (working alliance and real relationship), and more negative attitudes toward online therapy and its efficacy. After 3 months, perceived challenges with three domains – Emotional connection, Patients’ privacy, and Therapists’ boundaries significantly decreased – whereas challenges in the fourth domain – Distraction – increased. In our study, therapists’ concerns about being able to connect with patients online appeared to be the most impactful, in that it predicted negative attitudes toward online therapy and its perceived efficacy 3 months later, above and beyond the effect of therapists’ age and clinical experience. Clinical and training implications are discussed.

## Psychotherapists’ Challenges With Online Therapy During COVID-19: Concerns About Connectedness Predict Therapists’ Negative View of Online Therapy and Its Perceived Efficacy Over Time

In recent years, a body of pre-pandemic research has shown that online therapies’ and in-person therapies are comparable with regard to the quality of the working alliance (e.g., Simpson and Reid, 2014), and that online therapy can be similarly effective (Simpson, 2009; Backhaus et al., 2012). However, despite these empirical findings, therapists’ concerns about online therapy persisted and had hindered the uptake of online therapy via videoconferencing. In pre-pandemic times, many therapists, regardless of age and therapeutic orientation, were apprehensive about offering online therapy, and reluctant to integrate online therapy work into their regular practices. Most psychotherapists had little training and experience in providing online psychotherapy pre-pandemic, but nevertheless had multiple concerns about this therapy format.

For example, a major concern regarding online therapy in pre-pandemic times regarded the therapeutic relationship, as many therapists doubted the feasibility of building a strong therapeutic alliance in a remote setting (Roesler, 2017; Connolly et al., 2020). This concern has been found to correlate with a lack of intent to provide therapy online, however, as other studies pointed out, once therapists engaged in providing therapy online, this worry usually decreased (Sucala et al., 2013). Many psychotherapists were also worried about the online therapy relationship being impersonal and being less able to communicate empathy and emotions (Roesler, 2017). Concerns about the impact of technical glitches, insufficient Internet literacy, and confidentiality issues (e.g., Topooco et al., 2017; Titzler et al., 2018) and a belief that online treatment would be inferior to in-person therapy were also widespread (e.g., Topooco et al., 2017; for a review, see Connolly et al., 2020).

With the COVID-19 pandemic starting in early 2020, the involuntary mass transition to online therapy drastically changed this landscape. Following restrictions imposed to manage the COVID-19 pandemic, many therapists had to move their practice online, regardless of their previous attitudes and concerns about online therapy. Providing online therapy became an accepted necessity, and many therapists suddenly gained extensive experience with this therapy format.

According to pre-pandemic studies, first-hand experience with providing online therapy typically reduces or even diminishes concerns about its effectiveness (Reese et al., 2016; Tonn et al., 2017) and leads to more positive attitudes toward online psychotherapy than before (Donovan et al., 2015). It is thus possible that the experience of using online therapy during the pandemic might have a similarly positive impact on therapists’ concerns and attitudes toward online therapy. At the same time, mandatory versus voluntary use of technological innovations has been found to moderate the relationship between predicting factors and users’ attitudes (Venkatesh et al., 2003), and thus the involuntary nature of the transition to online therapy might have a negative impact on therapists’ attitudes on using online therapy. Moreover, the challenges of the abrupt transition, as well as the general stress associated with the global crisis situation might make online therapy during the pandemic a distressing experience for many therapists, from which they are unlikely to gain positive experiences or develop more positive views on online therapy (Messina and Loffler-Stastka, 2021).

Age has been theorized to also have an impact on attitudes toward technology use (Venkatesh et al., 2012), and younger age is often associated with being more technology savvy. In the context of online psychotherapy, pre-pandemic studies found no relationship between age and attitudes toward online therapy (Liu et al., 2015; Hennemann et al., 2017); however, therapists of all ages’ sudden and *en masse* transition to online therapy might have posed specific challenges to therapists based on their age and experience. That is, even though younger generations might have had more preliminary experience with video conferencing, which they might have used more for personal communication purposes, older, clinically more experienced therapists might have had the advantage of having developed more solid and transferable therapy skills that could be more easily adapted to the new online platform.

### Aims

With the present study, we aim to investigate therapists’ perceived challenges with providing online therapy during the pandemic, and to identify how these challenges shaped their experiences and attitudes toward online therapy during the pandemic. Our main research questions were threefold: (1) What are the main challenges experienced by therapists during the transition to online therapy and do these challenges differ among therapists of different ages and levels of experiences? What challenges did therapists experience 3 months later, and how did these experienced challenges change over time? (2) How do therapists’ perception of the quality of the relationship (working alliance and real relationship) with their online patients at the start of the pandemic relate to their perceived challenges at baseline and at the 3-month follow-up? (3) How do perceived challenges at the beginning of the pandemic and 3 months later relate to therapists’ attitudes toward online therapy and its efficacy at these two timepoints, when controlling for relevant covariates (age and experiences)?

## Materials and Methods

### Participants

As part of a large-scale international longitudinal online survey, we collected data from 1,257 therapists who provided at least one online therapy session since the beginning of the pandemic. Data were collected at two timepoints: first, between March and April 2020, after the global pandemic was declared and many therapists switched to online, and second, between June and July 2020, 3 months later. Recruitment details for this study have been reported previously (e.g., Aafjes-van Doorn et al., 2020a, b; Békés et al., 2020; Békés and Aafjes-van Doorn, 2020). The majority of the 1,257 therapists (77% female, mean age = 50.53, SD = 16.40) resided in North America, were licensed clinicians, and had 17 years or more of clinical experience. Many therapists had had no experience with providing online therapy before the pandemic, whereas others had seen patients for online sessions previously, but only after meeting them in-person first. A small minority of therapists had seen several patients for online treatment before the start of the pandemic. The majority of therapists did not have any training in how to provide online therapy. For further details on therapist characteristics, see Table 1.

**TABLE 1:** Descriptive characteristics of the therapists (*N* = 1257).

	*n*	*%*
**Location** North America Europe Australia Africa South America	1,029 159 7 4 3	82.0 12.7 0.6 0.3 0.2
**Licensure status** Licensed Trainee	1,076 181	86.1 23.9
**Years of clinical experience** 0–4 5–16 17 or more	159 465 633	12.7 36.9 50.4
**Previous experience of providing online therapy** No Yes, once or twice Yes several patients Yes, but only after seeing them in-person first	569 148 197 343	45.2 14.8 15.7 24.3
**Previous training in online therapy** Yes No	222 1,035	17.6 82.4

At both timepoints, therapists were asked about perceived challenges with switching from in-person to online therapy, acceptance of online therapy technology, and its efficacy compared to in-person sessions. At the first assessment point (i.e., baseline), we also assessed the therapists’ perception of the quality of the online therapeutic relationship (working alliance and real relationship). The sub-sample of participants who completed the 3-month follow-up measurement (*N* = 320) did not differ significantly on any demographic variables or in their attitudes and views on the efficacy of online therapy from the therapists who only provided baseline data.

### Measures

#### Therapist Challenges

All therapists were asked to report the challenges they experienced with conducting online therapy at baseline as well as at follow-up. “*During the pandemic, what are the main challenges for you using online therapy? (Multiple answers possible).*” Response options (yes or leave blank) were based on theoretical and clinical writings about challenges in online therapy pre-Covid and included the following 11 possible challenges: *Technical/internet problems, Difficult to read patient’s emotions, Difficult to feel/express empathy, Difficult to feel connected with the patient, Difficult to keep professional boundaries, Difficult for me to find a professional space for the online session, Difficult for the patient to find a suitable space for the online session, Confidentiality of the online sessions, Risk of me getting distracted during session, Risk of a patient getting distracted during session*, and *Scheduling is difficult.* Participants also had an option to report “other” challenges.

#### Working Alliance

The Working-Alliance Inventory-Short Form (WAI-SF; Hatcher and Gillaspy, 2006) for therapists was used to assess Bordin’s (1979) conceptualization of the working alliance, including the level of agreement on the goals of treatment, the therapeutic tasks, and the bond between the patient and the therapist. The WAI-SF consists of 10 items about the quality of the therapeutic relationship rated on a Likert scale ranging from 1 (never) to 7 (always). In in-person therapies, a WAI-SF rating of 4 (sometimes), the middle point of the scale, is interpreted as a neutral relationship, with no evidence in either positive or negative direction (Horvath and Greenberg, 1989). The WAI-SF has shown adequate reliability and validity (Hatcher and Gillaspy, 2006), useful in the prediction of treatment outcome (Munder et al., 2010; Zilcha-Mano, 2017). Cronbach’s alpha in the current study was 0.84 at baseline.

#### Real Relationship

The Real Relationship Inventory Therapist Form (RRI-T; Gelso et al., 2005) was used to assess the genuine human relationship between the patient and the therapist, an ongoing quality of the relationship that can be distinguished from transference and the working alliance (see the tripartite model; Gelso et al., 2018). The RRI consists of 24 items on a five-point Likert scale from Strongly Disagree (1) to Strongly Agree (5). Higher overall scores reflect a more genuine and authentic relationship. Research has indicated the reliability of the RRI to be high, with coefficient alphas ranging from 0.80 to 0.90 in various samples (e.g., Marmarosh et al., 2009; Fuertes et al., 2013, 2019). In this study, Cronbach’s α was 0.73.

#### Attitudes Toward Online Therapy

The Unified Theory of Acceptance and Use of Technology Therapist Version (UTAUT-T; Békés et al., manuscript underreview) was used to assess therapists’ attitudes toward online therapy technology. The UTAUT-T builds on the comprehensive model of acceptance and subsequent utilization of technological innovations (UTAUT framework; Venkatesh et al., 2003) that has previously been adapted to different professional contexts (Liu et al., 2015; Connolly et al., 2020; for a review, see Venkatesh et al., 2012).

The UTAUT-T consists of 21 items about various aspects of online therapy that are scored on a Likert scale ranging from 1 (strongly disagree) to 5 (strongly agree). For example, “I find online therapy works well for patients,” and “I feel apprehensive about using online therapy” (reverse item). Higher scores indicate a more positive attitude toward online therapy. Cronbach’s α was 0.64 in this study.

#### Perceived Online Therapy Efficacy

To examine how therapists perceived the efficacy of online therapy in comparison to in-person therapy, we included the following item to the baseline and follow-up survey; “How do you view online therapy now?” Responses were rated on a five-point Likert scale; *Definitely less effective than in-person therapy* (1); *Somewhat less effective than in-person therapy* (2); *As effective as in-person therapy* (3); *Somewhat more effective than in-person therapy* (4); and *Definitely more effective than in-person therapy* (5).

### Data Analytic Strategy

The analyses were based on baseline scores on the therapists’ perceived challenges, WAI, RR, and UTAUT-T, as well as the 3-month follow-up data on therapists’ perceived challenges and the UTAUT-T. To identify main challenges, we applied exploratory factor analysis using oblique genomin rotation to the 11 binary items in the perceived challenge questionnaire at baseline and 3-month follow-up. A robust weighted least squares estimator was used for binary indicators. We choose oblique rotations over orthogonal rotations as this seems to be more reasonable to assume factor correlations. Model fit statistics [chi-square (χ^2^); Bollen, 1989], comparative fit index (CFI; Bentler, 1990; values > 0.90 indicate acceptable fit), and root-mean-square error of approximation (RMSEA; Hu and Bentler, 1999, < 0.08 indicates acceptable fit) were used to identify the best factor structure, given their superior performance over parallel analyses in identifying factors with binary indicators (Finch, 2020). After identifying the best-fitting factor structure, responses of participants were re-coded based on whether they endorsed any challenges for each specific factor. Chi-Square tests were used to examine differences between endorsement of challenges at baseline and 3-month follow-up for the participants who provided both baseline and follow-up data.

To examine the relationship between perceived challenges and other variables and establish sufficient power, we calculated a continuous, mean score for each factor of perceived challenges for each therapist to be used in the correlation and regression analyses. We examined the Pearson correlations between scores for each factor of perceived challenges and relevant demographic variables (age and clinical experience), therapeutic relationship variables (WAI and RR), and attitudes and perceived efficacy of online therapy at baseline, while applying Bonferroni correlation for multiple testing. We also examined the concurrent correlation between perceived challenges and attitudes and perceived efficacy of online therapy at 3-month follow-up. Additionally, we conducted two linear regression models to examine whether perceived challenges at baseline predict attitudes and perceived efficacy of online therapy at the 3-month follow-up after controlling for age and clinical experience. Data were modeled with Mplus version 8.2 (Muthén and Muthén, 1998–2017) for exploratory factor analyses and with SPSS 25 for Pearson correlation and linear regression.

## Results

### Therapists’ Perceived Challenges at Baseline

Exploratory factor analysis of individual survey items at baseline and 3-month follow-up indicated a best-fitting model of four factors (model fit indices and factor loadings are shown in Tables 1, 2). The four different types of perceived challenges were: *Emotional connection* (therapists’ difficulties in feeling connected with patients, reading emotions, express or feel empathy), *Distraction* (therapist or patient getting distracted during sessions), *Patient privacy* (difficulties with confidentiality or in patient’s finding a private space for therapy), and *Therapist boundary* (therapists’ issues with creating a professional workspace and boundaries). The indicators for the items about technical/internet problems and for scheduling challenges did not load on any factors and were not associated with any of the outcome variables; therefore, they were removed from the subsequent analyses.

**TABLE 2:** Model fit indices for exploratory factor analyses of perceived challenges at baseline (*N* = 1,257) and 3-month follow-up (*N* = 320).

Model	# free parameters	χ^2^	df	*P*-value	CFI	RMSEA
Baseline						
One-factor	11	405.22	44	0.00	0.76	0.08
Two-factor	21	160.16	34	0.00	0.92	0.05
Three-factor	N/A*	–	–	–	–	–
**Four-factor**	**38**	**17.23**	**17**	**0.44**	**1.00**	**0.003**
Five-factor	45	7.24	10	0.70	1.00	0.000
3-Month follow-up						
One-factor	11	119.80	44	0.00	0.71	0.07
Two-factor	21	62.04	34	0.00	0.89	0.05
Three-factor	30	26.14	25	0.40	0.01	1.00
**Four-factor**^+^	**38**	**10.52**	**17**	**0.88**	**0.00**	**1.00**
Five-factor	45	3.69	10	0.96	0.00	1.00

The bolded model indicates the selected model for both timepoints. CFI, comparative fit index; RMSEA, root-mean-square error of approximation. P-value indicates the significance in the chi-square test for absolute fit.*Indicates that the three-factor model at baseline does not converge.^+^ Indicates that although the three-factor model in the 3-month follow-up has already reached an excellent fit, the four-factor structure for the follow-up data fits well with the four-factor structure identified at baseline; therefore, we chose the four-factor structure for data at both baseline and 3-month follow-up.

Among these four factors, Emotional connection, Distraction, and Patients’ privacy were endorsed as challenges approximately equally (52.3, 53.4, and 53.9%, respectively) at the beginning of the pandemic, whereas Therapists’ boundary issues were less of a concern (28.5%). About one-third of the therapists endorsed challenges in Technical/internal problems (68.8%) and only a small proportion reported challenges in Scheduling (6.9%).

Therapists’ age and clinical experience were significantly negatively related to all four types of challenges at baseline, that is, older and more experienced therapists perceived fewer challenges of any kind in their online sessions. These correlations remained significant after applying a Bonferroni correction for multiple testing. Results of the Pearson correlations are presented in Table 3.

**TABLE 3:** Pattern loadings for the four-factor dimensional model at baseline (*N* = 1,257) and 3-month follow-up (*N* = 320).

	Baseline				3-Month follow-up			
Item	Emotional Connection	Therapist Boundary	Distractions	Patient Privacy	Emotional Connection	Therapist Boundary	Distractions	Patient Privacy
1. Technical/Internet	−0.17	0.14	−0.04	0.09	−0.14	0.00	−0.03	0.38
2. Difficult to read patient’s emotions	**0.71**	−0.00	0.02	0.32	**0.72**	−0.07	−0.01	0.37
3. Difficult to express/feel empathy	**0.85**	0.01	−0.02	−0.06	**0.78**	0.02	−0.13	−0.01
4. Difficult to feel connected to patient	**0.67**	0.00	0.08	0.01	**0.72**	0.13	0.13	−0.03
5. Difficult to keep professional boundary	0.20	**0.35**	0.15	−0.04	0.09	**0.74**	0.05	0.05
6. Difficult to find a professional space	0.00	**0.73**	0.07	−0.04	−0.07	**0.67**	−0.06	0.30
7. Patient’s difficulty finding private space	−0.06	0.49	0.01	**0.40**	0.03	0.07	0.16	**0.66**
8. Confidentiality	0.26	0.37	−0.05	**0.30**	0.04	0.10	0.07	**0.49**
9. Risk of me getting distracted	0.02	0.07	**0.90**	−0.02	0.02	0.47	**0.40**	−0.29
10. Risk of patient getting distracted	−0.00	−0.02	**0.78**	0.62	−0.02	−0.02	**1.18**	0.03
11. Scheduling	0.09	0.17	−0.10	0.19	−0.18	0.26	−0.04	−0.06

*The bolded model indicates the loadings of the selected indicators for each factor at both timepoints.*

### Change in Therapists’ Perceived Challenges Over 3 Months

After 3 months, difficulties in Emotional connection, Distraction, and Patients’ privacy were endorsed at the rate of 49.4, 69.7, 51.9, and 22.2%, respectively, whereas the endorsement of challenges in Therapists’ boundary was at 22.2%. Difficulties with technical/internet problems and with scheduling were endorsed at 79.7 and 2.2%, respectively. At 3-month follow-up, age and clinical experiences were only related to difficulties with Therapists’ boundary and to Distractions, but not to Emotional connection and Patients’ privacy.

When comparing the endorsements of challenges at baseline and the 3-month follow-up for people who had data at both timepoints (*N* = 320), we found significant decreases in difficulties in Emotional connection (54.1% at baseline vs. 49.4% at follow-up, *χ^2^* = 63.7, *df* = 1, *p* < 0.001), Therapists’ boundary (28.7% at baseline vs. 22.2% at follow-up, *χ^2^* = 49.16, df = 1, *p* < 0.001), Patients’ privacy (60.0% at baseline vs. 51.9% at follow-up, *χ^2^* = 26.17, df = 1, *p* < 0.001), and Scheduling (6.9% at baseline vs. 2.2% at follow-up, *p* = 28.25, df = 1, *p* < 0.001). In contrast, we noticed significant increase in difficulties of Distraction (57.5% at baseline vs. 63.4% at follow-up, *χ^2^* = 47.25, df = 1, *p* < 0.001) and Technical/internet problems (68.8% at baseline vs. 79.7% at follow-up, *χ^2^* = 29.11, df = 1, *p* < 0.001). Thus, all perceived challenges seemed to have decreased 3 months after the start of the pandemic except for the increases in difficulties in Distractions of patients and therapists and in Technical/Internet problems.

### Perceived Challenges in Relation to Therapeutic Relationship Variables, and Attitudes and Perceived Efficacy of Online Therapy

At baseline, both working alliance and real relationship were significantly negatively related with challenges in Emotional connection (see Table 4). Working alliance was also significantly negatively related to challenges in Therapists’ boundaries. The real relationship at baseline was significantly related with challenges in Emotional connection at the 3-month follow-up, indicating that the genuineness of the therapeutic relationship was associated with less perceived challenges in Emotional connections 3 months later. Working alliance at baseline was significantly negatively related to challenges in Distraction at 3-month follow-up, indicating that poor working alliance at baseline may predict getting more distracted during online sessions 3 months later.

**TABLE 4:** Pearson correlations between categories of perceived challenges, demographic variables, therapeutic relationship, and attitudes and perceived efficacy of online therapy at baseline and 3-month follow-up.

	Age	Clinical experience	Real relationship	Working alliance	UTAUT	UTAUT at 3-month FL	Perceived efficacy	Perceived efficacy at 3-month FL
**Perceived challenges at baseline**								
Emotional Connection	**−0.20**	**−0.17**	**−0.21**	**−0.15**	**−0.44**	−0**.29**	**−0.38**	**−0.28**
Therapist Boundary	**−0.22**	**−0.22**	−0.04	**−0.11**	**−0.18**	−0.10	**−0.17**	−0.12
Patient Privacy	**−0.18**	**−0.13**	−0.08	−0.05	**−0.18**	−0.21	−0.09	−0.09
Distraction	**−0.25**	−**0.14**	−0.04	−0.09	**−0.15**	−0.12	−**0.11**	−0.10
**Perceived challenges at follow-up**								
Emotional Connection	−0.16	−0.14	−**0.19**	−0.18	−**0.29**	−**0.43**	−**0.24**	−**0.34**
Therapist Boundary	−**0.25**	−**0.30**	0.02	−0.08	−0.15	−0.07	−0.06	−0.05
Patient Privacy	−0.13	−0.16	−0.02	−0.05	−0.15	−0.11	−0.06	−0.06
Distraction	−**0.33**	−**0.32**	−0.19	−**0.19**	−0.16	−0.05	−0.07	−0.13

*The bolded coefficients indicated p < 0.001 (for Bonferroni correction p = 0.5/64 = 0.0008).*

Both attitudes and views on the efficacy of online therapy significantly improved from baseline to the 3-months follow-up assessment (*t* = −7.45, *p* < 0.001; *t* = −3.15, *p* < 0.01, respectively). At baseline, all types of challenges were associated with negative attitudes toward online therapy, and all but Patients’ privacy issues were associated with negative perceived efficacy of online therapy. However, only challenges with Emotional connection at baseline were negatively associated with attitudes and perceived efficacy of online therapy at 3-month follow-up. Similarly, only perceived challenges with Emotional connection (but not the other challenge categories) at follow-up remained associated with more negative attitudes toward online therapy and its efficacy at follow-up.

Two separate linear regressions were conducted to examine if the challenges reported at baseline predicted attitudes toward online therapy and its efficacy at follow-up, while controlling for the effect of age and clinical experience. Challenges with Emotional connection (*B* = −0.50, *SE* = 0.14, *t* = −3.54, *p* = 0.001) and challenges with Patients’ privacy (*B* = −0.30, *SE* = 0.13, *t* = −2.27 *p* = 0.03) at baseline predicted more negative attitudes toward online therapy at the 3-month follow-up (Δ*R*^2^ = 0.11), whereas age and clinical experience did not significantly contribute to these predictions. For perceived efficacy of online therapy, challenges with Emotional connection (*B* = −0.71, *SE* = 0.05, *t* = −13.10, *p* < 0.001) and challenges with Patients’ privacy (*B* = −0.23, *SE* = 0.06, *t* = −3.7, *p* < 0.001) at baseline predicted lower levels of perceived efficacy of online therapy at 3-month follow-up (Δ*R*^2^ = 0.15), whereas age and clinical experience did not significantly contribute to these predictions.

## Discussion

The COVID-19 pandemic provided a unique context in which to better understand therapists’ attitudes toward online therapy and how those attitudes change over time. In this study, we aimed to examine therapists’ perceived challenges with online therapy during the early months of the pandemic. More specifically, we examined the main challenges experienced by therapists during the transition from in-person to online therapy at the start of the pandemic and 3 months later, and their associations with therapists’ perception of the quality of the relationship with their online patients and their attitudes toward online therapy and its efficacy at these two timepoints.

Results indicated that initially many therapists reported multiple relational, technical, and practical challenges, and that overall, they reported fewer challenges 3 months later. Factor analysis of individual survey items at both timepoints indicated four different types of challenges: Emotional connection (difficulty with emotionally connect to the patient), Distraction (therapist or patient being distracted during sessions), Patients’ privacy (private space and confidentiality), and less frequently, Therapists’ boundaries (professional work space and issues with boundary setting). Therapists’ age and clinical experience were significantly negatively related to all four challenge categories, that is, older and more experienced therapists perceived fewer challenges in their online sessions. At the beginning of the pandemic, all four types of challenges were associated with lower perceived quality of the therapeutic relationship (working alliance and real relationship), and more negative attitudes toward online therapy and its efficacy.

After 3 months, perceived challenges with Emotional connection, Patients’ privacy, and Therapists’ boundaries significantly decreased, whereas challenges around Distractedness increased. Only perceived challenges with Emotional connection (but not the other three challenge categories) remained associated with more negative attitudes toward online therapy and its efficacy. Notably, therapists who reported a therapeutic relationship that was sufficiently genuine (high real relationship scores) early in the pandemic perceived less challenges regarding emotional connection 3 months later. Therapists who reported more challenges with Patients’ privacy issues at baseline subsequently perceived online therapy to be less efficacious. Challenges with Emotional connectedness at the start of the pandemic predicted more negative attitudes toward online therapy as well as its perceived efficacy 3 months later, whereas age and clinical experience did not significantly contribute to these predictions.

Our results indicate that therapists experienced multiple professional challenges during the early phase of the pandemic, and that these challenges were related to their experience of the quality of their therapeutic relationship, and their attitudes toward online therapy and its efficacy compared to in-person sessions both at baseline and 3 months later. The positive change in attitudes and views on the efficacy of online therapy, as well the overall decrease in perceived challenges reported over the 3 months align with pre-pandemic findings that concerns about online therapy typically decrease following the experience of using it (Connolly et al., 2020). Although accumulating evidence shows that the treatment efficacy is similar in online and in-person therapies (Marchand et al., 2011; Fernandez et al., 2021), at the beginning of the pandemic, therapists often did not have experience with the online therapy format, and lacked training and knowledge of its efficacy, which might have led to more negative initial views. Over time and with gaining more experience of practicing online therapy, these views became more positive.

In our study, participating therapists’ concerns about being able to emotionally connect with their patients online (express and feel empathy, feel connected, read patients’ emotions) appeared to be the most impactful; emotional connection predicted attitudes toward online therapy and its efficacy 3 months later, above and beyond the effect of therapists’ age and clinical experience. Therapists’ initial concern regarding connectedness in online sessions is in line with pre-pandemic studies that showed that connectedness with online patients is a major concern among therapists (Connolly et al., 2020), despite strong empirical evidence that the quality of working alliance is excellent in online therapy via videoconference (see review by Norwood et al., 2018), and comparable to in-person therapies (e.g., Bouchard et al., 2020; Watts et al., 2020).

These findings indicate that despite the forced and abrupt transition and the stress associated with the global crisis situation, psychotherapists had a reasonably good experience with online psychotherapy, in that many initial relational and practical challenges reduced over the first few months of the pandemic. This might be due to their ability to adapt to the new therapy format over time and finding ways to address the initially experienced challenges. The decrease in reported challenges can be understood by the therapists’ development of resilience, which reflects an adaptive process that unfolds over time (Bonanno, 2004; Chen and Bonanno, 2020). An exception was the experienced level of Distraction by therapists and patients, which appeared to increase over time. Studies in other contexts have shown that individuals are more prone to becoming distracted when using video conferencing compared to in-person settings (e.g., Brainard and Watson, 2020). Distraction reported by participants in this study may have been due to a decrease in alertness as therapists acclimated to the online platform. We could speculate that the increase in distraction may reflect various issues. First, during the pandemic, many aspects of our lives transitioned to online, which means that during an online therapy session, patients and therapists might have been distracted by messages, calls, and notifications that popped up on their device, reminding them of other commitments outside of therapy. Second, anecdotal evidence shows that self-view in video conferencing may also be distracting, as it diverges the attention to one’s appearance. Third, with the lack of commute to sessions, time spent in a waiting room, and a space for therapy which would be physically separated from the rest of everyday life, there is often no transitional space and thus opportunity to adjust one’s mindset between therapy and other aspects of life (such as work, doing chores at home, answering emails, etc.). Fourth, it is possible that distraction increases as the novelty of the online therapy platform wears off, requiring less focus and attention. Finally, the widespread phenomenon of “Zoom fatigue” likely contributed to a cumulative level of exhaustion impaired ability to focus (Bailenson, 2021). That said, these are speculations, as we did not ask participants about the exact nature of their experienced distractions at each timepoint. Future qualitative research is needed to better understand the nature of these distractions for patients and therapists over time.

Pre-pandemic studies show that challenges regarding connection emotionally with patients online have been a major concern among therapists (Roesler, 2017; Connolly et al., 2020). Our study shows, that despite of growing empirical evidence that the therapeutic alliance in online therapies is just as strong as in in-person settings (see review by Norwood et al., 2018), especially when rated by patients (e.g., Ruwaard et al., 2009), therapists still feel challenged by the relational aspects of online therapy, and this challenge has a significant and long-term impact on their attitudes and views on online therapy and its efficacy.

### Clinical and Training Implications

Acknowledging and addressing challenges regarding feeling and expressing empathy, feeling connected, and reading patients’ emotions in online sessions should now be a central part of therapist training, supervision, and continued education. Moreover, our results support the notion that first-hand experience with online therapy reduces negative attitudes toward online therapy and its efficacy. Our findings indicate that even under the stressful circumstances of a global pandemic and the involuntary transition to online therapy instead of in-person, experiencing the ability to overcome initial challenges and personally learning what it is like to provide online therapy led to more positive attitudes toward online therapy and its efficacy. Although at this point even the short-term future of online therapy is uncertain, an important implication of these results is to expose psychology trainees to the online therapy format early enough so that they can learn skills of how to manage challenges related to online therapy, and to experience its benefits. Training of junior therapists appears to be especially important in this regard, given that in our study, younger and less experienced therapists appeared to be more challenged by the switch to online therapy compared to their older and more clinically experienced counterparts.

### Limitations

Several limitations may be noted. First, although the diverse therapists’ sample increases the relevance of its findings for therapists in different treatment settings and across the world, it cannot be interpreted as representative of all online therapists globally. There might be several unexamined therapist demographics that influence the therapists’ perceived challenges and attitudes toward online therapy, such as the nature of the therapists’ professional network, other relevant professional and personal experiences during and before COVID-19, technical and emotional resources, and patient-demand. Second, given the relatively large sample size more advanced statistical analyses using machine learning models might have been usefully applied, to explore therapists’ differences in relation to the assessed variables (see Aafjes-van Doorn et al., 2021). Third, this survey study did not examine the experienced challenges of participating therapists’ respective patients. Patients’ attitudes are important to examine, especially because the online interventions are designed for and paid by patients and might have been especially crucial in this time of global distress. Therapists might be willing to encounter challenges as part of their professional duties; however, patients have more at stake, as they are the people who pay for services and their treatment outcomes might be ultimately affected. The patients’ experienced challenges during this forced transition to online therapy are thus arguably the most important. Future studies might benefit from a 360-degree perspective on the online therapy experiences, including viewpoints from patients and clinical supervisors. Moreover, the survey responses on therapists’ challenges and perceived efficacy of online therapy were single items that, although based on theory and clinical writings, were not part of standardized measures. The development of standardized scales of experienced challenges in (transitioning to) online therapy as well as its perceived efficacy is warranted. Finally, this empirical study applied only quantitative measures, and thus did not provide contextual insights into the specific therapists’ circumstances. Future research designs may benefit from the inclusion of a qualitative approach and might be able to tease apart what parts of the therapists’ experiences resulted from the unique situation of COVID-19 and what part reflects online therapy more generally.

## Conclusion

All in all, the COVID-19 pandemic has provided a unique context in which to examine therapists’ perceived challenges in providing online therapy, not just based on preconceived worries but based on involuntary extensive experience. Therapists in our study struggled with connecting emotionally with patients, getting distracted during sessions, ensuring adequate patient privacy, and with maintaining their own boundaries in sessions. These challenges initially led to less positive views on the therapeutic relationship, and on online therapy and its efficacy, but typically diminished over time, except that therapists became more easily distracted in online sessions over time. Concerns about being able to connect with patients were the most impactful, as it predicted negative attitudes toward online therapy and its perceived efficacy over time. Overall, therapists’ views on online therapy and its effectiveness become more positive over time.

Results will need to be replicated in online therapy sessions outside of the pandemic, as it is possible that the societal unrest and high-stress context influenced therapists’ perceived professional challenges and openness to new technologies. More research and professional training is needed to address the challenges faced by therapists when transitioning to online therapy, especially around the ability to emotionally connect with patients online and how to manage distractibility that is inevitable in an online therapy format.

## Data Availability Statement

The data analyzed in this study are subject to the following licenses/restrictions: The datasets may be available on request. Requests to access these datasets should be directed to VB, vera.bekes@yu.edu.

## Ethics Statement

The studies involving human participants were reviewed and approved by the Yeshiva University’s Western IRB. The patients/participants provided their written informed consent to participate in this study.

## Author Contributions

VB and KAD contributed to collecting the data and writing the manuscript. XL contributed to the data analysis. TP and LH contributed to collecting the data and finalizing the manuscript. All authors contributed to the article and approved the submitted version.

## Conflict of Interest

The authors declare that the research was conducted in the absence of any commercial or financial relationships that could be construed as a potential conflict of interest.
